# Changes in spinal motoneuron excitability during the improvement of fingertip dexterity by actual execution combined with motor imagery practice

**DOI:** 10.1016/j.heliyon.2024.e30016

**Published:** 2024-04-23

**Authors:** Yuki Fukumoto, Marina Todo, Makoto Suzuki, Daisuke Kimura, Toshiaki Suzuki

**Affiliations:** a Kansai University of Health Sciences, Faculty of Health Sciences, Department of Physical Therapy, 2-11-1 Wakaba Sennangun Kumatori, Osaka, 590-0482, Japan; b Graduate School of Kansai University of Health Sciences, Graduate School of Health Sciences, 2-11-1 Wakaba Sennangun Kumatori, Osaka, 590-0482, Japan; c Bukkyo University, Faculty of Health Sciences, Department of Occupational Therapy, 7 Higashitochio-cho Nishinokyo Nakagyo-ku, Kyoto, 604-8418, Japan; d Nagoya Women's University, Faculty of Medical Science, Department of Occupational Therapy, 3-40 Shioji Mizuho Nagoya, Aichi, 467-8610, Japan

**Keywords:** Motor learning, Repetition training, F-wave, F/M amplitude ratio, Force adjustment task, Motor skill

## Abstract

Since there is an upper limit to skill improvement through the repetition of actual execution, we examined whether motor imagery could be used in combination with actual execution to maximize motor skill improvement. Fingertip dexterity was evaluated in 25 healthy participants performing a force adjustment task using a pinch movement with the left thumb and index finger. In the intervention condition, six sets of repetitions of combined actual execution and motor imagery were performed, while in the control condition, the same flow was performed, but with motor imagery replaced by rest. Changes in the excitability of spinal motoneurons during motor imagery compared to rest were compared in terms of the F/M amplitude ratio. Motor skill changes were compared before and after repeated practice and between the conditions, respectively, using the absolute amount of adjustment error between the target pinch force value and the delivered pinch force value (absolute error) as an index. The results showed that the repetition of exercise practice and motor imagery decreased the absolute error, which was greater than that of exercise practice alone in terms of motor skill improvement. The F/M amplitude ratio for motor imagery compared to rest did not increase. This suggests that motor imagery is involved in the degree of the increase of spinal motoneuron excitability based on the real-time prediction of motor execution and that there may be no need for an increase in excitability during motor skill control.

## 1 Introduction

When motor skills are lost due to some pathological condition, e.g., stroke, actual execution (AE) must be repeated to relearn motor skills. Motor learning is defined as a series of processes based on practice and experience that result in a relatively permanent change in the ability to perform a skilled behavior [1]. Although the repetition of AE is generally considered effective for improving motor skills, it is also known that a ceiling effect exists, limiting the effect of AE that can be achieved. For example, Miyaguchi et al. [2] examined motor skill changes over time during 10 sets of repetition of a right index finger force adjustment task. They found that repetitive AE was associated with a significant increase in motor skill over time. They also reported that repeated AE contributed to motor skill improvement, but most of the skill improvement was observed by the sixth AE session, with no significant improvement between the seventh and tenth sessions. Thus, to maximize the efficiency of motor learning, strictly defined as the retention of motor skills, which is different from changes in motor skill observed immediately after practice [3], it is necessary to achieve an immediate change after AE with the highest possible accuracy.

An intertrial interval is the period during which the motor program is modified by taking into account errors from the previous exercise performance [4]. Motor imagery (MI) is a cognitive process that utilizes working memory and is defined as the reproduction of motor-related memories [5]. Therefore, repeated MI practice (MIP) of performance-enhancing performed during the intertrial interval can be used to enhance the modification of the exercise program, and several reports have found the combination of AE and MIP to be useful [6]. Specifically, the mechanism by which the combined use of AE and MIP is effective is thought to be that the combination of bottom-up and top-down effects results in the simultaneous strengthening of higher-level circuits. That is, AE causes changes in central brain regions as a result of bottom-up effects from peripheral body regions [7], while MIP causes changes as a result of top-down effects from motor preparation regions in the central brain to motor and peripheral body regions [8]. Indeed, the combination of AE and MIP reportedly improves upper limb dysfunction [9] and hand function [10] compared to AE alone. However, there are some reports that the combination of AE and MIP has no specific effect [11,12]. This discrepancy is suggested to be because the methods for the implementation of MIP were not specified [6], and a certain consensus has not yet been reached on whether the combination of AE and MIP is truly useful or for the methods for implementing MIP.

We have continued to examine the effective practices for applying MIP alone, since MIP is thought to contribute to motor skill improvement because it shares the same neural basis as motor execution [13], and the consideration of MIP-induced motor skill changes should be discussed along with the neurophysiological background. There is a certain consensus that the neurophysiological background during MIP involves the activation of central brain regions involved in motor execution, primarily in the supplementary motor cortex [[14], [15], [16], [17], [18]]. However, only a few reports have focused on regulation at the spinal level, and contradictory results have been presented, with some reports showing no change in excitability [19,20] and others showing an increase [21,22]. Since excitability at the spinal cord level, especially spinal motoneurons, is the final common pathway of motor commands directly related to motor expression and it also explains the decrease in precise pinch force control skills [23]. In our previous studies examining this issue, MIP improved motor skills related to hand precision and increased spinal motoneuron excitability [24], but we have also found conflicting results, with increased spinal motoneuron excitability, but no improvement in motor skills [25]. This suggests that an increase in spinal motoneuron excitability does not lead to an improvement of motor skill related to hand accuracy, but rather that the adjustment of spinal gain conditions [26]. In support of this, study focusing on MIP strategies showed that the execution of highly precise MIP and improved hand precision motor skills, although the excitability of spinal cord motor neurons was not altered [27]. Thus, we hypothesized that combining AE and MIP with strict adherence to MIP rules would prevent the overexcitation of spinal motoneurons during MIP, resulting in higher motor skill levels than with AE alone.

The novelty of this study was to examine the combined effect of AE and MIP with strict MIP implementation rules and to track motor skill changes in light of excitability changes in spinal motoneurons. For this purpose, we clarified whether the combination of AE and MIP is useful in healthy participants, excluding individual factors such as the presence of disease that might be expected to interfere with the experimental results.

## 2 Materials and methods

### 2.1 Participants

G*power software (version 3.1.9.4) was used for analysis of variance in repeated measures and within factors. In this study, it was necessary to compare the differences in motor skills derived from the results of a series of AE and MIP combination processes and to capture changes over time during the combination practice in terms of changes in the spinal motoneuron excitability. Therefore, since two statistical methods are used in this study, a power analysis was conducted for each. The type of power analysis was set to “*a priori*: compute required sample size-given α, power, and effect size.” The sample size estimation considered the possibility of high effect sizes in this study because our previous studies [17] showed a high effect size. The effect size *f* was set to 0.4, α error probability was set to 0.05, power (1-β error probability) was set to 0.8, the number of groups was set to 1, the number of measurements was set to 6, correlation among repetitive measures was set to 0.5, and nonsphericity correction epsilon was set to 0.2. The sample size was calculated to be 23. A paired *t*-test was used when the data were normally distributed, and the Wilcoxon signed-rank test was used when the data were not normally distributed. The types of power analysis used were “*a priori*: compute required sample size given α, power, and effect size,” “difference between two dependent means (matched pair),” “Wilcoxon signed-rank test (matched pair).” The effect size *dz* was set to 0.8, α error probability was set to 0.05, and power (1-β error probability) was set to 0.8. The sample size was calculated to be 15. Considering participant dropout, 25 healthy participants who self-reported as being right-handed were included to exceed the appropriate sample size in both settings (13 males and 12 females, mean age 23.8 ± 6.1 years). Twenty-five participants were assigned to both the intervention and control conditions as the corresponding sample. The participants were fully informed of the significance and purpose of the study in accordance with the Declaration of Helsinki and written informed consent was obtained before measurements were taken (Kansai University of Health Sciences Research Ethics Review Committee, Ethics No.: 22-04).

### 2.2 Experimental set up

**Fig. 1:**
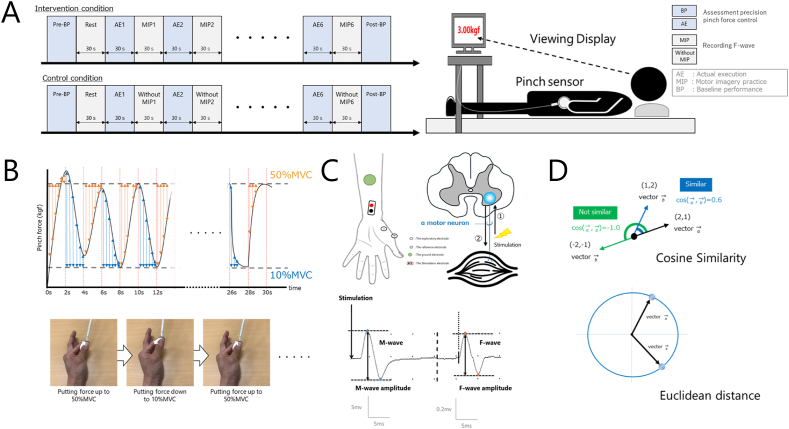
Experimental procedures (A) The flow of the study, with the intervention condition performing six repetitions of a combination of actual execution and motor imagery, and the control condition performing six repetitions of a combination of actual execution and rest. The blue boxes assess motor skills, and the gray boxes assess the excitability of spinal motoneurons. (B) Description of the motor task, in which the participant is required to quickly and accurately adjust pinch force alternately between 50 % and 10 % MVC using an audible cue that is sounded at 2-s intervals. During the motor task, the exerted pinch force value (kgf) can be confirmed visually in real time. (C) Derivation of F waves, an index of spinal motoneuron excitability. The stimulating electrode is affixed on the median nerve at the left wrist joint, the probing electrode is affixed on the muscle group on the left thumb ball, the reference electrode is affixed on the dorsal surface of the left first metacarpal head, and the ground electrode is affixed on the central palm side of the left forearm. As a result of electrical stimulation from the stimulating electrode, action potentials are conducted retrogradely through motor nerve fibers (①). Next, after re-firing in the axonal subiculum of the anterior horn cells of the spinal cord, the action potential is conducted in a forward direction, and F waves are obtained from the dominant muscle as a compound muscle action potential (②). (D) Schematic illustration of two indicators of similarity. The upper row is the cosine similarity, which represents the cosine value of the angle formed by two vectors in vector space. The lower row is the Euclidean distance, which evaluates similarity in terms of the shortest linear distance between two points.

The experiment was conducted in a controlled laboratory at 25 °C. The participants were placed in a comfortable supine position throughout the experiment. First, fingertip dexterity was assessed with a pinch task using the thumb and index finger of the left hand to determine baseline performance (Pre-BP). Next, changes in the excitability of spinal motoneurons were measured during 30 s of rest in the supine position (Rest). When AE and MIP are used together, the effect is more likely to be obtained if MIP is inserted in the inter-trial interval after AE [6], so the order of implementation was to perform 30 s of AE, assess fingertip dexterity during AE, and then perform 30 s of MIP practice in the intervention condition. In MIP, the participants were asked to recall the content of the previous AE and alternated between “imagine the contraction of the thumb ball muscle during the pinch movement” and “imagine the numerical increase or decrease in pinch force displayed on the pinch meter” as an imagery strategy [24]. In the control condition, the participants did not perform MIP (without MIP). During MIP and without MIP, changes in spinal motoneuron excitability were measured in six sets of repetitions. Fingertip dexterity was again assessed with a pinch task using the left thumb and index finger (Post-BP). The order in which each condition was performed was randomized, with a 1-week washout period (Fig. 1A).

### 2.3 Fingertip dexterity task

The motor task was an alternating force adjustment task in which the participants adjusted the pinch force to 50 % maximum voluntary contraction (MVC) and 10 % MVC at 2-s intervals, and the pinch force was displayed in real-time on a pinch meter (Digital Indicator F340A; Unipulse, Inc.). The task was performed while the participants visually monitored the displayed pinch force. The timing of the pinch force switch was controlled by an auditory stimulus at 2-s intervals using a metronome. The index of fingertip dexterity was calculated as the absolute error from the specified value by subtracting the 50 % MVC (or 10 % MVC) value set as the specified value from the participant's exerted pinch force, converting the error value into an absolute value, and then averaging the absolute error during the 30-s motor task (total performance). The unit of absolute error used as a measure of Fingertip dexterity was kgf. In addition, 50 % MVC performance, which is an excerpt of the data from the 50 % MVC adjustment, and 10 % MVC performance, which is an excerpt of the data from the 10 % MVC adjustment, were also calculated. Fingertip dexterity was measured with EMG recording software (Vital Recorder2; KISSEI COMTEC Co., Ltd.) during the Pre- and Post-BP periods and each AE task and analyzed using a versatile bioanalysis system (BIMUTAS-Video; KISSEI COMTEC Co., Ltd.) (Fig. 1B).

### 2.4 Spinal motoneuron excitability

Viking Quest ver. 9.0 (Natus Medical, Inc.) was used with a band frequency of 20 to 3 kHz and sampling frequency of 10 kHz. F waves were used as an index to evaluate the changes in spinal motoneuron excitability, which are complex muscle action potentials recorded in the corresponding muscles as retrograde impulses generated by maximal electrical stimulation of peripheral mixed nerve fibers that travel up and then down the axon as progressive impulses as a result of re-firing in anterior horn cells of the spinal cord. The F wave recording conditions were as follows: the stimulating electrode was placed on the median nerve at the left wrist joint, the exploration electrode was placed on the muscle group on the left thumb ball, the reference electrode was placed on the dorsal surface of the left first metacarpal head, and the ground electrode was placed on the palmar side of the left forearm. A bipolar bar electrode (Au) was used as the stimulating electrode, a 10-mm diameter EEG cup electrode (Ag/AgCl) was used as the reference/exploration electrode, and a 30-mm diameter plate ground electrode (stainless steel) was used as the ground electrode (Fig. 1C). F-wave analysis was based on the F/M amplitude ratio, which reflects the increase in the number of muscle fibers that re-fire in the anterior horn cells of the spinal cord [28]. The F/M amplitude ratio was measured during rest and MIP (or without MIP).

### 2.5 Examination of MIP implementation

After all experiments were completed, two surveys were conducted to determine how the participants performed MIP. First, the participants were asked to judge each MIP session on a 6-point Likert scale ranging from 0 (not at all difficult) to 5 (very difficult and unimaginable) in terms of the difficulty of performing MIP. Next, the participants were asked to respond in an open-ended format to what they were imagining during MIP of adjusting to 50 % or 10 % MVC.

### 2.6 Data analysis

Since the Shapiro-Wilk test showed non-normality in the data, a non-parametric test was used. First, absolute errors were examined using Wilcoxon's signed-ranked test for within-condition changes in the Pre- and Post-BP periods. Comparisons between the two conditions were also made using the Wilcoxon signed-rank test for normalized data considering each subject's Pre-BP motor capacity (Subtract the Pre-BP from the Post-BP). A two-way analysis of variance with a generalized linear mixed model was conducted for both conditions (Intervention/Control) and number of AE trials (first to sixth) to see whether the addition of MIP enhances motor skill improvement during AE trials. In addition, cosine similarity and Euclidean distance were calculated and compared to determine whether the pattern of change in total performance over time was strongly influenced by changes in either 50 % or 10 % MVC performance using the Wilcoxon's signed-ranked test. Cosine similarity refers to the cosine value of the angle between two vectors in vector space (total performance and 50 % MVC performance/total performance and 10 % MVC performance). For two vectors, $\vec{x}=\left(x_{1},x_{2},\cdots x_{n}\right),\vec{y}=\left(y_{1},y_{2},\cdots y_{n}\right)$, cosine similarity is defined as follows:

$$\cos\left(x,y\right)=\frac{\left\langle x,y\right\rangle }{\left\|x\right\|\left\|y\right\|}=\frac{\sum_{k=1}^{n}x_{k}y_{k}}{\sqrt{\sum_{k=1}^{n}x_{k}^{2}}\sqrt{\sum_{k=1}^{n}y_{k}^{2}}}$$

Note that the closer cos(x,y) is to 1, the higher the similarity between $\vec{x}$ and $\vec{y}$. It is important to note that it only represents the relationship between vector orientations and is not affected by the magnitude of the vectors. Next, the L2 norm, also called Euclidean distance, was expressed as follows using the three-square theorem when the *d*-dimensional vector $x=\left(x_{1},x_{2},\cdots x_{d}\right)$:

$${\left\|x\right\|}_{2}=\sqrt{\sum_{i=1}^{d}{\left|x_{i}\right|}^{2}}$$

Therefore, by subtracting the Euclidean distance at 50 % MVC performance from total performance and subtracting the Euclidean distance at 10 % MVC performance from total performance, then comparing these values, a more similar MVC can be found (Fig. 1D).

Next, we performed multiple comparisons of the F/M amplitude ratio during MIP1 to MIP6 (or without MIP1 to without MIP6) for changes in spinal motoneuron excitability, using Rest as a control, with the Steel test. In addition, Spearman's correlation coefficients were used to examine the relationship between the absolute error in total performance at each AE and the change in the F/M amplitude ratio during each MIP (or without MIP) session concerning Rest.

Finally, using MIP1 as a control, we performed multiple comparisons of the difficulty of performing MIP from MIP2 to MIP6 with the Steel test. The significance levels were all set at less than 5 %, and the effect size (r) was calculated based on the Z value for Wilcoxon's signed-ranked test. SPSS ver. 26.0 (IBM, Inc.) and the open-source software R (4.1.0) were used as statistical analysis software.

## 3 Results

### 3.1 Fingertip dexterity

**Fig. 2:**
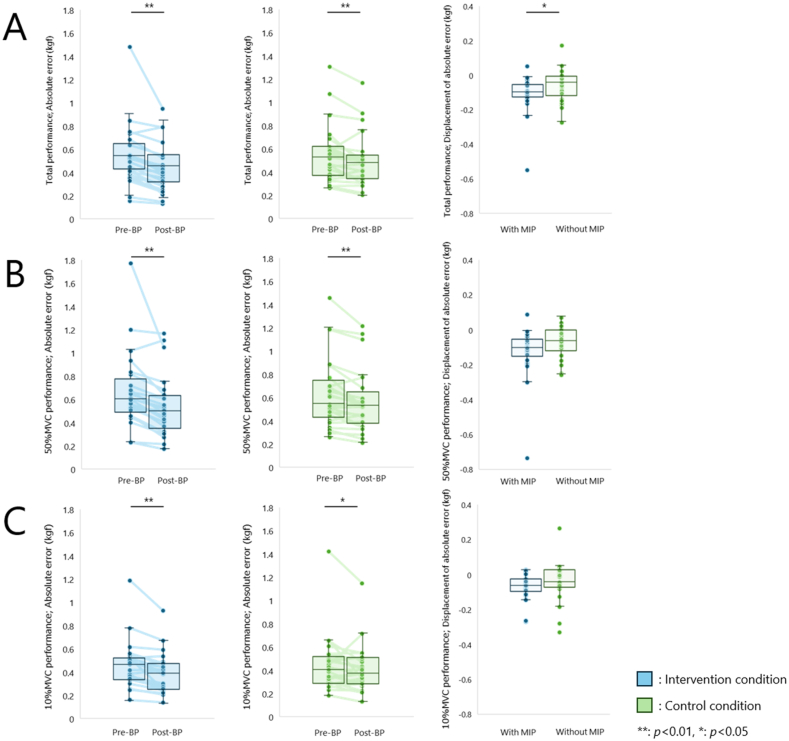
Motor skill changes (A) Blue and green boxes indicate the intervention and control conditions, respectively. Total performance, in which motor skills improved after repetition in both conditions. Furthermore, motor skills after repetition of actual execution and motor imagery were at a higher skill level than after repetition of actual execution alone. (B) 50 % MVC performance, in which motor skills improved after repetition in both conditions. However, motor skills after repetition of actual execution and motor imagery were not different from those after repetition of actual execution alone. (C) 10 % MVC performance, in which motor skills improved after repetition in both conditions. However, motor skills after repetition of actual execution and motor imagery were not different from those after repetition of actual execution alone.

**Fig. 3:**
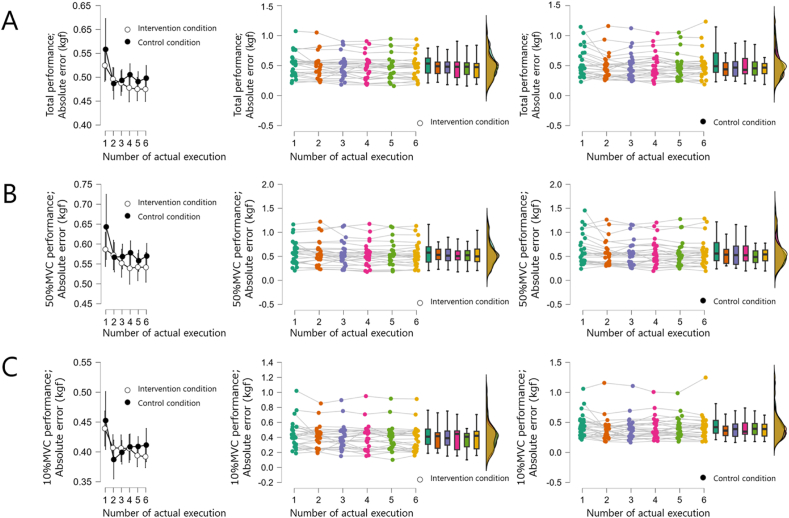
Changes in motor skills during actual execution The insertion effect of motor imagery practice seemed to emerge after a series of actual execution was accomplished, and no change was observed during motor practice. (A) Total performance, no interaction was observed. (B) 50 % MVC performance, no interaction was observed. (C) 10 % MVC performance, no interaction observed.

Total performance for the intervention condition was 0.55 (0.44–0.60) kgf for Pre-BP, 0.53 (0.38–0.63) kgf for AE1, 0.49 (0.37–0.56) kgf for AE2, 0.48 (0.38–0.56) kgf for AE3, 0.48 (0.30–0.65) kgf for AE4, 0.48 (0.35–0.55) kgf for AE5, 0.47 (0.30–0.53) kgf for AE6, and 0.46 (0.33–0.52) kgf for Post-BP compared to Pre-BP, indicating a decrease in absolute error (*p* < 0.001, r = 0.84). Total performance for the control condition was 0.53 (0.38–0.62) kgf for Pre-BP, 0.49 (0.39–0.71) kgf for AE1, 0.44 (0.34–0.55) kgf for AE2, 0.47 (0.32–0.57) kgf for AE3, 0.43 (0.36–0.62) kgf for AE4, 0.45 (0.34–0.56) kgf for AE5, 0.47 (0.37–0.53) kgf for AE6, and 0.48 (0.35–0.54) kgf for Post-BP. The absolute error decreased for Post-BP compared to Pre-BP (*p* = 0.003, r = 0.59). However, in an inter-condition comparison that considered exercise capacity based on each subject's Pre-BP (intervention condition, −0.09 (−0.12–0.05) kgf; control condition, −0.04 (−0.10–0.01) kgf), the absolute error in the intervention condition was further reduced compared to the control condition (*p* = 0.045, r = 0.40 (Fig. 2A). On the other hand, no interaction was observed in the change in absolute error in the AE trial in each condition (F (5,264) = 0.535, *p* = 0.749 (Fig. 3A)).

The 50 % MVC performance for the intervention conditions was 0.60 (0.51–0.72) kgf for Pre-BP, 0.58 (0.38–0.70) kgf for AE1, 0.53 (0.43–0.65) kgf for AE2, 0.51 (0.41–0.65) kgf for AE3, 0.50 (0.43–0.61) for AE4, 0.52 (0.41–0.62) kgf for AE5, 0.50 (0.39–0.65) kgf for AE6, and 0.50 (0.37–0.62) kgf for Post-BP. The absolute error decreased in Post-BP compared to Pre-BP (*p* < 0.001, r = 0.83). The 50 % MVC performance for the control condition was 0.55 (0.45–0.71) kgf for Pre-BP, 0.56 (0.41–0.79) kgf for AE1, 0.54 (0.35–0.65) kgf for AE2, 0.53 (0.33–0.71) kgf for AE3, 0.52 (0.40–0.72) for AE4, 0.49 (0.37–0.61) kgf for AE5, 0.54 (0.41–0.63) kgf for AE6, and 0.53 (0.40–0.60) kgf for Post-BP, with a decreased absolute error in Post-BP compared to Pre-BP (*p* < 0.001, r = 0.67). However, in an inter-condition comparison that considered exercise capacity based on each subject's Pre-BP (intervention condition, −0.10 (−0.13–0.05) kgf; control condition, −0.06 (−0.10–0.01) kgf), no difference was found between the control and intervention conditions (*p* = 0.128, r = 0.30 (Fig. 2B). Furthermore, there was also no interaction effect on the change in absolute error in the AE trial in each condition (F (5,264) = 0.578, *p* = 0.716 (Fig. 3B)).

The 10 % MVC performance for the intervention condition was 0.46 (0.34–0.52) kgf for Pre-BP, 0.41 (0.31–0.51) kgf for AE1, 0.42 (0.25–0.46) kgf for AE2, 0.39 (0.30–0.49) kgf for AE3, 0.45 (0.24–0.49) for AE4, 0.41 (0.27–0.45) kgf for AE5, 0.42 (0.25–0.48) kgf for AE6, and 0.39 (0.25–0.47) kgf for Post-BP. The absolute error decreased in Post-BP compared to Pre-BP (*p* < 0.001, r = 0.80). The 10 % MVC performance for the control condition was 0.41 (0.29–0.50) kgf for Pre-BP, 0.42 (0.32–0.53) kgf for AE1, 0.36 (0.28–0.44) kgf for AE2, 0.39 (0.27–0.47) kgf for AE3, 0.35 (0.30–0.49) for AE4, 0.39 (0.30–0.48) kgf for AE5, 0.39 (0.27–0.47) kgf for AE6, and 0.37 (0.28–0.50) kgf for Post-BP, with a decrease in absolute error in Post-BP compared to Pre-BP (*p* = 0.037, r = 0.42). However, in an inter-condition comparison that considered exercise capacity based on each subject's Pre-BP (intervention condition, −0.06 (−0.08–0.02) kgf; control condition, −0.04 (−0.06–0.02) kgf), no difference was found between the control and intervention conditions (*p* = 0.109, r = 0.32 (Fig. 2C). Furthermore, there was also no interaction effect on the change in absolute error in the AE trial in each condition (F (5,264) = 0.535, *p* = 0.749 (Fig. 3C),).

Changes over time in total performance in the intervention condition were influenced by changes in the ability to adjust to 50 % MVC performance in both cosine similarity and Euclidean distance (cosine similarity: *p* = 0.001, r = 0.65; Euclidean distance: *p* < 0.001, r = 0.86). Changes in total performance in the control condition over time were also affected by changes in the ability to adjust to 50 % MVC performance in both cosine similarity and Euclidean distance (cosine similarity: *p* < 0.001, r = 0.78; Euclidean distance: *p* < 0.001, r = 0.86) (Table 1).

**Table 1:** Euclidean distance by cosine similarity and L2 norm. The left side shows the intervention condition and the right side shows the control condition, both of which were influenced by the degree of improvement in 50 % MVC performance in total performance.

	With MI condition								Without MI condition							
	Cosine Similarity				Euclidean distance				Cosine Similarity				Euclidean distance			
	Total×50%MVC	Total×10%MVC	*p* value	effect size(r)	Total－50%MVC	Total－10%MVC	*p* value	effect size(r)	Total×50%MVC	Total×10%MVC	*p* value	effect size(r)	Total－50%MVC	Total－10%MVC	*p* value	effect size(r)
N1	0.999634543	0.996941279	*p* = 0.001	r = 0.65	−0.342022053	0.396085247	*p* < 0.001	r = 0.86	0.997867953	0.999497838	*p* < 0.001	r = 0.78	−0.036344700	0.086429038	*p* < 0.001	r = 0.86
N2	0.998717871	0.997803464			0.009306729	0.012858576			0.998240732	0.998212650			0.025221303	−0.008897382		
N3	0.999881644	0.999613578			−0.716725669	0.818495368			0.999116084	0.999063360			0.013191259	−0.016185243		
N4	0.999958059	0.999070264			−0.602044621	0.719101197			0.999473471	0.999368160			−0.122234906	0.133003160		
N5	0.999827220	0.998981213			−0.829257506	0.986215259			0.999822182	0.999615295			−0.055982406	0.078090992		
N6	0.999695849	0.999329959			−0.109441066	0.180540800			0.999653537	0.999274113			−0.141699346	0.167921144		
N7	0.985784923	0.996327144			−0.256383724	0.245904129			0.999120644	0.998567166			−0.118971261	0.163452788		
N8	0.998575678	0.997236441			−0.083725808	0.108816210			0.999374879	0.998717282			−0.073558368	0.138431904		
N9	0.998635417	0.994322524			−0.271222613	0.340291837			0.999897912	0.999228962			−0.907999482	1.071376930		
N10	0.999628709	0.999161746			−0.084007607	0.099899119			0.999235827	0.998508381			−0.077954410	0.137736007		
N11	0.998917430	0.995308549			−0.236140723	0.269996379			0.999707138	0.998826049			−0.356264872	0.408911951		
N12	0.998898740	0.994820264			−0.076232443	0.109283052			0.998570145	0.997595080			−0.083577568	0.105002642		
N13	0.997590002	0.994159306			−0.231997404	0.286027595			0.999840811	0.998756425			−0.782802503	0.923132528		
N14	0.999549067	0.999271421			−0.066184070	0.087097680			0.999257997	0.998081547			−0.231452350	0.274690709		
N15	0.999741105	0.999697430			−0.038320849	0.054683370			0.998229277	0.996898129			−0.152513256	0.184470247		
N16	0.999795071	0.998680147			−0.114678569	0.138916717			0.999198534	0.997769994			−0.125283080	0.159942844		
N17	0.999434955	0.997024816			−0.165012046	0.200387684			0.999355884	0.997618322			−0.152555041	0.211959351		
N18	0.998043460	0.998662924			−0.060244339	0.139331344			0.998926742	0.997103455			−0.130100748	0.186712863		
N19	0.999360992	0.997038129			−0.146637404	0.193293513			0.999440441	0.997281288			−0.148251272	0.193213165		
N20	0.998980906	0.998890117			−0.087603112	0.125812523			0.998976565	0.996597125			−0.280576780	0.353315620		
N21	0.999165644	0.999476619			0.033942868	−0.023203514			0.999620489	0.996772964			−0.631313768	0.782809803		
N22	0.999463167	0.998913571			−0.091519652	0.137838016			0.998747223	0.987758381			−0.641895415	0.721799860		
N23	0.999616739	0.999240208			−0.222919985	0.251703983			0.999513429	0.999126823			−0.058376558	0.095511350		
N24	0.999559266	0.998786776			−0.113923812	0.166357372			0.999158185	0.997666286			−0.055530346	0.077353763		
N25	0.999776539	0.999179942			−0.071876851	0.099035909			0.998620011	0.996472507			−0.059606154	0.025769761		

### 3.2 Spinal motoneuron excitability

**Fig. 4:**
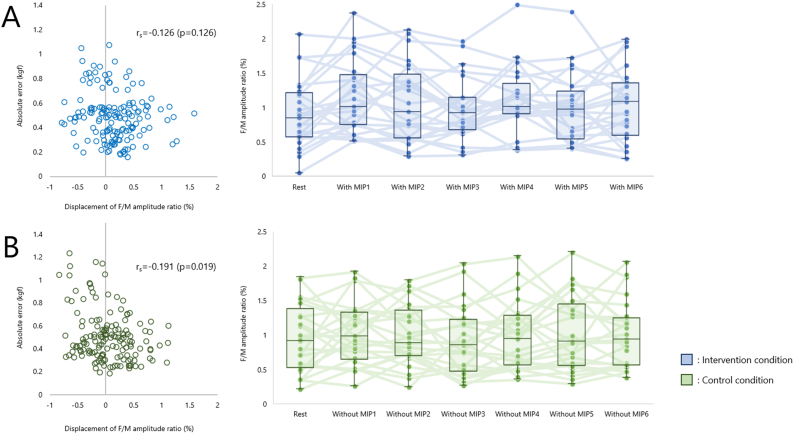
Excitability changes in spinal motoneurons (A) Intervention condition. There was no difference in the F/M amplitude ratio during each motor imagery session compared to rest. (B) Control condition. There was no difference in the F/M amplitude ratio during each motor imagery session compared to rest. In addition, the relationship between motor skills during each actual execution session and the F/M amplitude ratio during no motor imagery showed no correlation (Spearman's rank correlation coefficient).

The F/M amplitude ratio for the intervention condition was 0.84 (0.58–1.19) % for Rest, 1.01 (0.75–1.43) % for MIP1, 0.93 (0.56–1.39) % for MIP2, 0.92 (0.69–1.14) % for MIP3, 1.01 (0.92–1.24) % for MIP4, 0.97 (0.59–1.17) % for MIP5, and 1.08 (0.62–1.33) % for MIP6, with no difference between each MIP session with Rest as a control (vs. MIP1: *p* = 0.344; vs. MIP2: *p* = 0.892; vs. MIP3: *p* = 0.968; vs. MIP4: *p* = 0.379; vs. MIP5: *p* = 0.984; vs. MIP6: *p* = 0.887). There was no correlation (r_s_ = −0.130, *p* = 0.114) between the absolute error (kgf) in each AE session for total performance and the F/M amplitude ratio change (%) with respect to Rest (Fig. 4A).

The F/M amplitude ratio for the control condition was 0.91 (0.55–1.28) % for Rest, 0.98 (0.66–1.28) % for without MIP1, 0.88 (0.70–1.29) % for without MIP2, 0.85 (0.50–1.18) % for without MIP3, 0.94 (0.60–1.26) % for without MIP4, 0.90 (0.55–1.38) % for without MIP5, and 0.93 (0.57–1.21) % for without MIP6; no difference was found between each without MIP session and Rest as a control (vs. without MIP1: *p* = 0.983; vs. without MIP2: *p* = 0.994; vs. without MIP3: *p* = 1.000; vs. without MIP4: *p* = 0.999; vs. without MIP5: *p* = 0.996; vs. without MIP6: *p* = 0.994). There was no correlation (r_s_ = −0.191, *p* = 0.019) between the absolute error (kgf) in each AE session and the F/M amplitude ratio change (%) with respect to Rest (Fig. 4B).

### 3.3 Implementation of MIP

**Fig. 5:**
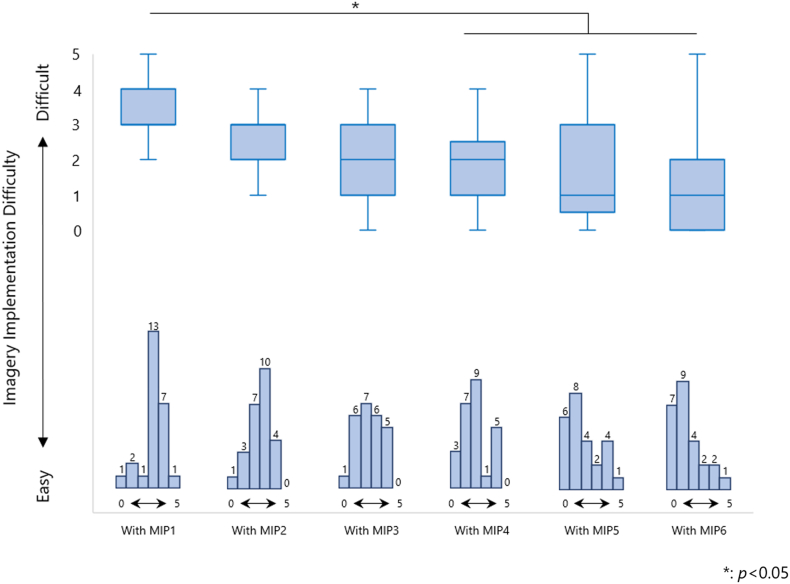
Difficulty of performing motor imagery. The degree of difficulty in performing motor imagery, and the box-and-whisker diagram and histogram showing the percentage of responses are shown below. The difficulty of performing motor imagery decreased from the fourth time onward, compared to the first time.

The difficulty of performing MIP from MIP2 to MIP6, with MIP1 as a control, showed a decrease from MIP4 to MIP6 (vs. MIP2: *p* = 0.188; vs. MIP3: *p* = 0.103; vs. MIP4: *p* = 0.010; vs. MIP5: *p* = 0.011; vs. MIP6: *p* < 0.001) (Fig. 5). Table 2 shows the open-ended responses of what the participants were thinking while performing MIP for adjusting to 50 % or 10 % MVC.

**Table 2:** Free descriptions of motor imagery content. The left side shows the adjusted motor imagery of 50 % MVC and the right side shows the adjusted motor imagery content of 10 % MVC, and each answer from the participants is listed as it was described.

	What did you consciously imagine in the process of having the force adjusted to 50%MVC?	What did you consciously imagine in the process of having the force adjusted to 10%MVC?
N1	Gradually exerted force and aimed at 50 % value	Gradually relaxing, I aimed for a value of 10 %
N2	To apply force more strongly	To pinch lightly
N3	How to apply force to the thumb	than 50 %, while relaxing the thumb while putting more force into it
N4	Remembering the amount of force when you actually worked on it	Remembering the amount of force when you actually worked on it
N5	Image of mother and index finger strongly together	Imagine slowly releasing the thumb and index finge
N6	Numbers	Numbers
N7	Muscle contraction and the number	Numbers
N8	Do not relax too much	Image as if you hold it lightly
N9	The number	The number
N10	Image of muImage of muscle contraction	Imagine that the muscles relax
N11	So that the nails turn white	How much force can be weakened
N12	Image of gradual contraction of the ball of the thumb	Image of gradually relaxing the thumb ball
N13	Grip stronger than you think	Grip the pinch meter just enough not to drop it
N14	The amount of force	How much you can weaken the force
N15	Muscle contraction during actual adjustment	Sense of relaxing the whole body
N16	Image of applying force	Image of relaxing
N17	Muscle contraction	Relaxation of muscles
N18	Muscle contraction	Imaged to bring the strength to 10%MVC while relaxing
N19	Image of exerting force strongly	Image of 10 % strength softly
N20	Contraction of the muscle	Do not relax too much
N21	Put in about 60 % of the force	Put in about 15 % strength
N22	Putting in the force	I was able to do it well from the beginning, I need to be able to keep it up
N23	Do not overdo it	It's like being in a sauna to get in shape
N24	Imagine pinching something delicate	I'm going to put some strength in my fingertips
N25	Try to grip it a little tighter	Let's relax the hands a little bit

## 4 Discussion

In the present study, we found that absolute error (kgf), a measure of motor skill, did not decrease linearly with each successive AE session, but rather fluctuated between sessions, i.e., increasing, decreasing, or remaining constant, until finally reaching a higher skill level. However, the combination of AE and MIP showed less variation due to the increase or decrease in errors, and ultimately resulted in higher skill levels than with AE alone. This motor skill change was particularly influenced by the degree of improvement in 50 % MVC performance, suggesting that MIP had some interference effect, but the repetition of MIP decreased the difficulty of performing MIP. There was no increase in the F/M amplitude ratio during each MIP session compared to Rest, nor was there any correlation with absolute error.

### 4.1 Improvement of motor skill through the repetition of AE with MIP

The forms of learning involved in motor skill improvement include “error-based learning,” “reinforcement learning,” “use-dependent learning,” and “cognitive strategies,” and although their relative contributions vary with the motor task, they are not completely separate and are thought to achieve skill acquisition in an interrelated manner [29]. Error-based learning results in behavior modification based on error signals that compare the kinesthetic consequences of expected and realized movements [30], while reinforcement learning involves learning and selecting appropriate motor behaviors by using prediction errors encoding the probability that the behavior will be successful [31]. Furthermore, in use-dependent learning, motor memories are formed by changing the current movement to resemble the previous one (goal-independent) in a repetition of the same movement [32], while in cognitive strategies, individuals develop new strategies to perform a behavior successfully [30]. In other words, use-dependent learning can be advanced by simply repeating an exercise without depending on a goal, but in situations in which motor skill improvement is required, some exploration is said to occur during repetition of the exercise. This explains why motor skills do not improve linearly, but are explored through the repetition of variable motor output. Exploration is considered useful for immediate skill improvement, and faster learning rates are observed with higher task-related variability both when only success-reward information is presented (reinforcement learning) and when error information is also presented (error-based learning) [33]. Furthermore, intentionally generated variability in motor output as a trial-and-error process (planned noise) can reportedly produce new motor control policies (cognitive strategies) for motor activities that improve performance and reduce costs [34].

In the present study, with six repetitions of AE, the absolute error (kgf) did not decrease progressively in both conditions, but rather fluctuated between increasing, decreasing, and remaining constant, leading to skill improvement. Therefore, it is suggested that the examined motor skill may have improved as a result of a certain degree of exercise repetition in both conditions. However, based on the results of this study, which examined the combination of AE and MIP, the possibility that MIP amplified these learning components during AE is considered.

As for the effect of MIP on each learning component, we evaluated the amount of learning in a visuomotor transformation task based on the change in angle when the participants were told that their movement was actually off-target, but were stopped just before movement execution. This is similar to MIP in that the movement was recalled, but not performed. Learning was found to have progressed even in the absence of motion execution, suggesting that MIP may be involved in error-based learning [35]. Furthermore, neurofeedback strategies using beta-band frequencies during MIP with brain-machine interfaces reportedly allow for the improvement of hand precision through dynamic threshold adaptation based on reinforcement learning [36]. Although these are reports showing the effects of MIP being amplified by the combined use of MIP rather than MIP alone, we believe that the present study, which examined the combined effects of AE and MIP, supports the possibility that MIP amplified these learning components during AE. The position of MIP insertion may also have been important in the present study; when AE and MIP are used together, it is easier to observe an effect if MIP is inserted after AE [6]. In the present study, MIP was performed after AE, and the intertrial interval between the end of AE and the start of the next AE is considered to be the point at which the exercise program is modified by considering errors in the memory of the previous exercise performance [4]. It is now clear that motor learning proceeds even when MIP is performed alone, and imagining finger movements displaced 90° from those produced by transcranial magnetic stimulation increases motor-evoked potentials in areas of the primary motor cortex corresponding to 90° displaced finger movements after imagery [37]. In other words, even MIP alone may facilitate motor learning by involving use-dependent learning. Furthermore, it has been shown that prefrontal regions, especially the dorsolateral prefrontal cortex, are activated during MIP in addition to motor-related areas [18], and the dorsolateral prefrontal cortex may play an important role in scenarios in which cognitive strategies need to be developed to solve the demands of a task. It is thought that the dorsolateral prefrontal cortex plays an important role in scenarios that require the development of cognitive strategies to solve task demands, and in particular, the higher the initial adaptation rate of learning, the higher the level of activation [38]. Thus, it is possible that MIP conducted during the intertrial interval may have been involved in the acquisition of higher motor skill levels, either as a stand-alone effect of MIP or as an amplification of the AE effect.

Conversely, in terms of Euclidean distance by cosine similarity and L2 norm, the improvement in motor skills in the alternating force adjustment task in both conditions was roughly influenced by the degree of improvement in 50 % MVC performance. This suggests that the characteristics of the alternating force adjustment task may be adjusted differently depending on target contraction strength. In the first place, in this study, the participants had to learn two different types of force adjustment using the same movement. In this regard, it has been reported that when conflicting perturbations are presented alternately for the same movement in a reaching movement task in a force field where the direction of the perturbation is reversed, learning is hindered, but substantial learning occurs with the use of MIP [7]. Thus, MIP may promote motor learning even if different elements are included in the same physical exercise. This is based on the property that MIP is potentially leveraged as a simulator, which can isolate and engage motor memory as multiple different motor memories, even when rehearsing the same physical movement [39].

On the basis of the above, it is possible that the control condition, in which MIP was not performed, had a greater improvement component in 50 % MVC performance [40], which is the more easily focused on of the two types of force coordination performance. However, this gradient continued in the intervention condition in which MIP was performed, with Post-BP between-condition comparisons showing changes in total performance, but no difference in 50 % and 10 % MVC performance. The lack of improvement in only one of the two in the studies from which either 50 % MVC or 10 % MVC was excerpted suggests that the improvement in motor skills limited to one ability did not affect overall performance, but rather the sum of the two. However, since overall performance was similar to the 50 % MVC results, it appears that it is the 50 % MVC that is helping to improve motor skills relative to the other. The relatively small impact of the change in 10 % MVC performance can be attributed to the MIP content of the 10 % MVC adjustment. The most common responses from the participants were “lessening force” and “relaxing.” Relaxation imagery is less likely to be effective in healthy participants [41]. Furthermore, for a competence adjustment task to 50 % MVC, it is considered sufficient to perform MIP for 1 min or less [42], but for relaxation imagery, 2 min are required [43]. In conclusion, the relaxation imagery used during the adjustment imagery to 10 % MVC was unlikely to be effective due to the perspective that the participants were healthy and the time period allowed for its implementation, so it is thought that the improvement in 50 % MVC performance was still relatively easy to recognize.

### 4.2 Changes in spinal motoneuron excitability during repetitive MIP

The excitability of spinal motoneurons during MIP is regulated via descending fibers from central brain regions [17,18,22]. Since changes in spinal motoneuron excitability during MIP are the result of adjustments in spinal gain conditions in preparation for subsequent motor performance [26], it is important to determine whether increased excitability of spinal motoneurons is necessary based on predictions of subsequent motor task performance. With regard to changes in excitability at the level of the spinal cord during motor control, the H-reflex amplitude of the radial carpometacarpal flexor muscle is reportedly decreased after learning sequential upper limb movements [44] and that the F/M ratio is also decreased with skill control, even in relatively difficult motor tasks [45]. Therefore, the motor skill improvement associated with repeated AE sessions suggests that the excitability of spinal motoneurons during MIP did not need to show an increase based on the prediction of motor execution immediately afterward.

The results so far show the effects of interference with MIP due to motor task characteristics and skill maturation, but it is thought that MIP implementation itself also influences the outcome. Although it includes the speculative aspect of piecing together the results of each indicator, the difficulty level of MIP changed with repetition, and after the fourth repetition, its difficulty level decreased compared to the first repetition. MIP reportedly increases spinal motoneuron excitability when its difficulty is high, but not when its difficulty is low [46]. Furthermore, when simple MIP of low difficulty is performed continuously, participants become accustomed to performing the imagery itself and the excitability of spinal motoneurons does not increase [47]. A difference in motor skill improvement between conditions was observed in Post-BP after all repetitions were completed, which we believe is a result of the reduced difficulty of MIP caused by its repetition and the habituation of the participants to MIP implementation, which facilitates the benefits of the MIP effect and ultimately leads to motor skill improvement.

Next, differences in MIP strategies may also have influenced the results. Similar to the present study, increased spinal motoneuron excitability was described in a report in which participants were asked to perform kinesthetic imagery in a force adjustment task for hand pinch movements [48]. Conversely, while kinesthetic imagery is useful for improving motor skills [49], it has also been noted to be difficult to implement [50]. However, it has been shown that by combining the recall of information related to the motor task (numerical information) in addition to the implementation of kinesthetic imagery, it is possible to implement MIP with high accuracy, thus facilitating the improvement of motor skills and eliminating the need to increase the excitability of spinal motoneurons [27]. From the perspective of MIP strategy, it is possible that the present study, in which the participants were asked to recall information related to the motor task (numerical information) in addition to kinesthetic imagery, resulted in highly accurate MIP and did not increase spinal motoneuron excitability. It has also been shown that the ability to accurately perform distinct MIPs without difficulty occurring is necessary for motor skill improvement, for example, if distinct MIPs can be performed without difficulty barriers or mental effort, excitability of spinal motor nerve function is not increased [51]. Therefore, as shown in this study, it seems that it is the inhibitory process that could intervene when performing distinct MIP that lead to motor skill improvement.

### 4.3 Limitations

The strength of this study is that it was able to suggest that a combination of AE and MIP could be a useful therapeutic tool, especially in light of changes in spinal motoneuron excitability that have not been consistently reported. On the other hand, it was difficult to observe improvement in motor skills in a small portion of the participants. Whether this is a matter of personal factors or whether there are still concerns about the overall method of implementation is a topic for further study. It has also been reported that learning in a fatigued state negatively affects overall task mastery [52] that repeated practice of AE and MIP induces peripheral and central fatigue, and that this fatigued state interferes with motor skill acquisition [53]. Furthermore, it has been reported that mental fatigue is readily observed during the repeated practice of AE and MIP, especially during MIP execution, and that fatigue decreases the MEP amplitude during MIP [54]. In addition, since one of the objectives of this study was to compare two conditions, AE with and without MIP, it is possible that each subject's motor imagery ability may have made a difference in the beneficial effects of MIP. This could be confirmed by assessment methods such as the VMIQ-2 [55], which was not validated in this study. Therefore, it is possible that the fatigue associated with MIP execution did not increase the excitability of spinal motoneurons, and the degree of fatigue associated with repetitive execution should be asked, and furthermore, the failure to consider motor imagery ability is a limitation of this study.

## 5 Conclusion

This study was to examine the combined effect of AE and MIP with strict MIP implementation rules and to track motor skill changes in light of excitability changes in spinal motoneurons. In the intervention condition, six sets of repetitions of combined actual execution and motor imagery were performed, while in the control condition, the same flow was performed, but with motor imagery replaced by rest. The combination of AE and MIP showed less variation due to the increase or decrease in errors, and ultimately resulted in higher skill levels than with AE alone. There was no increase in the F/M amplitude ratio during each MIP session compared to the Rest, nor was there any correlation with absolute error. Thus, it is possible that MIP conducted during the intertrial interval may have been involved in the acquisition of higher motor skill levels, either as a stand-alone effect of MIP or as an amplification of the AE effect. It has also been shown that the ability to accurately perform distinct MIPs without difficulty occurring is necessary for motor skill improvement, for example, if distinct MIPs can be performed without difficulty barriers or mental effort, excitability of spinal motor nerve function is not increased [51]. Therefore, as shown in this study, it seems that it is the inhibitory process that could intervene when performing distinct MIP that lead to motor skill improvement.

## Funding

This research was supported by the Meiji Yasuda Health and Welfare Foundation's 38th Health Science Research Grant for Young Researchers and by an Incentive Research Grant from 10.13039/501100004044Kansai University of Health Sciences.

## Ethical approval

The participants were fully informed of the significance and purpose of the study in accordance with the Declaration of Helsinki and written informed consent was obtained before measurements were taken (Kansai University of Health Sciences Research Ethics Review Committee, Ethics No.: 22-04).

## Data availability statement

The data that support the findings of this study are available from the corresponding author upon reasonable request.

## CRediT authorship contribution statement

**Yuki Fukumoto:** Writing – review & editing, Writing – original draft, Project administration, Methodology, Investigation, Funding acquisition, Formal analysis, Data curation. **Marina Todo:** Writing – review & editing, Methodology, Investigation, Data curation. **Makoto Suzuki:** Writing – review & editing, Formal analysis. **Daisuke Kimura:** Writing – review & editing, Formal analysis. **Toshiaki Suzuki:** Writing – review & editing, Formal analysis.

## Declaration of competing interest

The authors declare the following financial interests/personal relationships which may be considered as potential competing interests:Yuki Fukumoto reports article publishing charges was provided by Meiji Yasuda Life Foundation of Health and Welfare.
